# Ionic interactions of copper(ii) sulfate in water, aqueous maltose, and aqueous lactose at different temperatures: a volumetric and thermodynamic study

**DOI:** 10.1039/d5ra03403e

**Published:** 2025-09-24

**Authors:** Syed Muhammad Saqib Nadeem, Rehana Saeed, Moattar Ali

**Affiliations:** a Department of Chemistry, University of Karachi Room No. 309, 3rd Floor Karachi-75270 Pakistan smsaqibnadeem@gmail.com

## Abstract

Densities of pure water, aqueous maltose, and aqueous lactose solvents were measured at temperatures of 303.15, 308.15, 313.15, 318.15, and 323.15 K before and after the addition of different concentrations of copper(ii) sulfate. The density of water, aqueous maltose, and aqueous lactose solvents increased with the addition of copper(ii) sulfate, while an increase in temperature decreased the density. The apparent molar volume (*V*_ϕ_) of copper(ii) sulfate solutions was calculated from the density data by using a standard mathematical relationship. The *V*_ϕ_ decreased with the increase in the concentration of copper(ii) sulfate and is also affected by the nature of the solvent and temperature. The limiting apparent molar volume (*V*^0^_ϕ_) was evaluated by using Masson's and Redlich, Rosenfeld, & Meyer's equations. The structure-breaking or structure-making behavior of copper(ii) sulfate was evaluated by calculating Hepler's constant (δ^2^*V*^0^_ϕ_/δ*T*^2^)_*P*_. A negative value of Hepler's constant in water and aqueous maltose solvent confirmed the structure-breaking behavior of copper(ii) sulfate, whereas a positive value of Hepler's constant in aqueous lactose solvent is indicative of the structuring of the solvent. The limiting apparent molar expansibility (*E*^0^_ϕ_) and isobaric thermal expansion coefficient (*α*_p_) were also evaluated from the *V*_ϕ_ to provide supporting evidence for the obtained conclusions.

## Abstract 2

Densities of pure water, aqueous maltose, and aqueous lactose solvents were measured at temperatures of 303.15, 308.15, 313.15, 318.15, and 323.15 K before and after the addition of different concentrations of copper(ii) sulfate.

## Graphical abstract

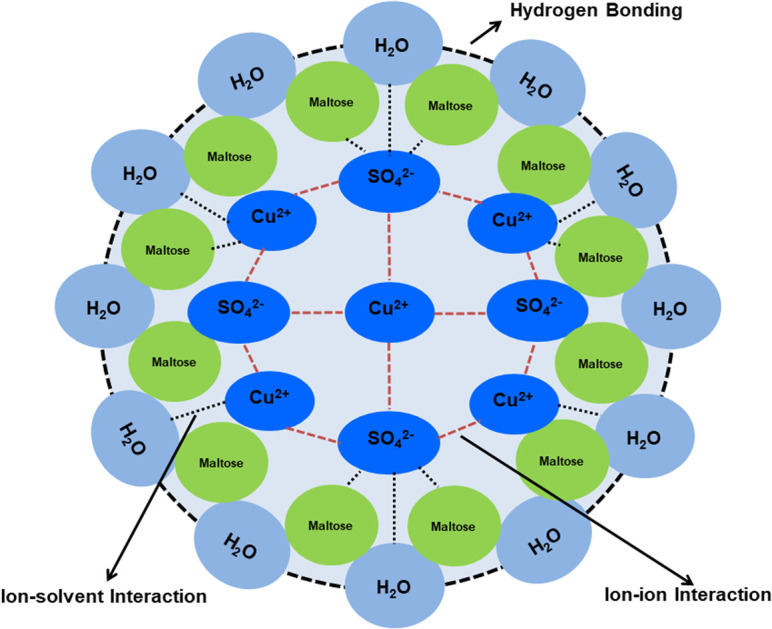

## 1. Introduction

Carbohydrates are a class of organic compounds having a general chemical formula C_*x*_(H_2_O)_*y*_; hence, they are given the name hydrated carbons.^1^ Maltose and lactose are water-soluble disaccharides that often exist in a monohydrated form, having a white crystalline appearance. Maltose and lactose have the same molar mass, but they differ in monomeric units, chemical bonding, conformational structure, and dipole moment. Maltose and lactose are primary sources of energy for the human body, and salt–sugar solutions are used as an instant source of energy and electrolytes for the human body, especially during exhaustive situations like dehydration, surgical procedures, and sports.^2^ In recent times, the research focus has been shifted toward the industrial applications of carbohydrates in nutraceutical supplements, therapeutic tools for drug delivery, biomedical materials, pharmaceutical formulations, bioactive substances, prebiotics, and laboratory diagnostic kits.^3–8^ Carbohydrates such as cellulose, keratin, and fibroin are used for the synthesis of textile fibers, whereas starch, lignin, and chitosan are increasingly used in the production of biodegradable plastics due to their non-cytotoxic behavior and environmentally friendly nature.^9,10^ Cellulose is used in the paper industry, whereas the polysaccharide extracts of red algae are an essential constituent of many cosmetics.^11^ Recently, the quest for the development of renewable fuels and energy sources has led to the utilization of carbohydrates as feedstocks for biofuels and industrial chemicals due to their economic feasibility and abundance in the ecosystem.^12–16^

The concentration of copper in the human body is the third highest among the trace elements after iron and zinc.^17^ Copper is an essential food mineral for humans because of its role as a catalyst in the synthesis of hemoglobin, iron metabolism, functioning of the nervous system, immune cells, sugar metabolism, and biosynthesis of proteins.^18–20^ The deficiency of Cu^2+^ in the human body can often result in slow growth and behavioral disorders, especially in children.^21^ The industrial applications of copper(ii) sulfate are as an additive in frozen and/or packaged foods in trace quantities, as a pesticide in agriculture, as a dye fixative in tannery, as an antifouling agent in paints, and as a raw material in the paper industry.^22^

The knowledge of volumetric and thermodynamic properties is critical for the design of technological processes, and the storage and transportation of products in the chemical, nuclear, and leather industries.^23,24^ The measurement of density is a very sensitive and accurate tool for evaluating the volumetric and thermodynamic properties in aqueous and non-aqueous solvents and the nature of ion–ion and ion–solvent interactions in complex electrolyte solutions.^25–32^ Maltose and lactose are a part of the daily human diet; therefore, a comprehensive understanding of the nature of the interaction of these disaccharides with water and electrolytes from the perspective of volumetric and thermodynamic changes is vital in evaluating their behavior inside the human metabolic system. Much literature is available on the ionic interactions of different electrolytes in water and aqueous maltose solvent by density measurement, but studies on the interaction of salts with lactose in an aqueous medium are very rare, especially from the perspective of structural differences between different disaccharides.^33–38^ This research work is aimed at investigating the structural changes in the bulk water by the addition of maltose/lactose and copper(ii) sulfate and evaluating the physical parameters such as limiting apparent molar volume (*V*^0^_ϕ_), limiting apparent molar volume of transfer (Δ_t_*V*^0^_ϕ_), limiting apparent molar expansibility (*E*^0^_ϕ_), and isobaric thermal expansion coefficient (*α*_p_) for the interaction of these two co-solutes with each other in pure water as a solvent to simulate the interaction of these essential components of human diet inside the human metabolic system in the context of solute–solute and solute–solvent interactions by using density measurements as an investigative tool. The structure-making or structure-breaking behavior of copper(ii) sulfate in water and the effect of the structural differences such as the nature of chemical bonding, conformational structure, and polarity between maltose and lactose on the interaction of copper(ii) sulfate with water is determined by analyzing the variation in the limiting apparent molar volume (*V*^0^_ϕ_) concerning temperature by evaluating the Hepler's constant whereas the thermodynamic parameters of the electrostatic interactions are also evaluated to provide insight into the volumetric changes associated with the ion-solvation process.

## 2. Materials and methods

The specifications of the chemicals used in the experimental work are provided in Table 1. The glassware used for the preparation of copper(ii) sulfate solutions was of Pyrex A-grade quality (Iso-Lab, Germany) with a tolerance of ±0.1%. The 1.0%, 3.0%, and 5.0% w/v concentrations of aqueous maltose and aqueous lactose solvents were prepared by dissolving the required amount of solid maltose and lactose in double-distilled water at ambient temperature. The copper(ii) sulfate solutions with concentrations of 1.0, 3.0, 5.0, 7.0, and 9.0 × 10^−2^ ± 4.0 × 10^−5^ (mol kg^−1^) were prepared by dissolving the required amount of copper(ii) sulfate crystals in the solvents. The required temperature of the solutions to be analyzed was achieved by keeping the solvents and solutions in a calibrated thermostatic water bath (Model YCM-01, Taiwan) having a least count of 0.1 °C for 10 minutes. A relative density bottle of 25.0 cm^3^ capacity with an uncertainty of 0.05 cm^3^ was used to measure the mass of a fixed volume of solvent and solution by an electronic weighing balance (ATX 224 Shimadzu, Japan) having an accuracy of ±0.01 mg. The density values of the solvent and solution were used to calculate the apparent molar volume of copper(ii) sulfate solutions in water, aqueous maltose, and aqueous lactose solvents at temperatures of 303.15, 308.15, 313.15, 318.15, and 323.15 K. The observations were recorded in triplicate, and the uncertainty in the experimental results is expressed along with each value or data set.

**Table 1:** The specifications of chemicals^a^

Chemical	Source	Purification method	Final purity (%)	Purity measurement method
**Copper(** **ii** **) sulfate**	E. Merck, Germany	Nil	99.98%	Purity was mentioned on the packaging
(Blue Crystalline Solid; AR Grade)				
Molar mass: 249.69 g mol^−1^				
Density: 2.29 g cm^−3^				
**Maltose** (White Crystalline Solid; AR Grade)	E. Merck, Germany	Nil	99.99%	Purity was mentioned on the packaging
Molar mass: 360.32 g mol^−1^				
Density: 1.54 g cm^−3^				
**Lactose** (White Crystalline Solid; AR Grade)	E. Merck, Germany	Nil	99.99%	Purity was mentioned on the packaging
Molar mass: 360.32 g mol^−1^				
Density: 1.55 g cm^−3^				
**Double distilled water** (Freshly Distilled Water)	Indigenously prepared	Double distillation	0.06 μS cm^−1^	Conductivity measurement
Molar mass: 18.02 g mol^−1^				
Density at 30 °C: 0.99565 g cm^−3^				

a Copper(ii) sulfate is toxic and a mild irritant, so it should be handled with gloves.

## 3. Results and discussion

The density measurements and volumetric analyses were used to evaluate the magnitude and nature of ionic interactions in the copper(ii) sulfate solutions in water and different concentrations of aqueous maltose and aqueous lactose solvents. The results are presented in Tables 2–11.

**Table 2:** Densities of pure water, aqueous maltose, and aqueous lactose solvents at different temperatures and ambient pressure^a^

Solvent	Density (*d*) (g cm^−3^)				
	303.15 K	308.15 K	313.15 K	318.15 K	323.15 K
Water	0.99565	0.99403	0.99222	0.99021	0.98804
1.0% aqueous maltose	1.01730	1.01400	1.01200	1.00900	1.00700
3.0% aqueous maltose	1.03510	1.02950	1.02670	1.02100	1.01570
5.0% aqueous maltose	1.05300	1.04800	1.04235	1.03313	1.02422
1.0% aqueous lactose	1.03640	1.03400	1.03201	1.03000	1.02500
3.0% aqueous lactose	1.04230	1.04040	1.03811	1.03670	1.03410
5.0% aqueous lactose	1.06002	1.05501	1.04932	1.04300	1.03915

a The uncertainty in the density data of solvents is in the range of ±0.2%.

**Table 3:** Densities of copper(ii) sulfate solutions at different temperatures and ambient pressure^a^

[CuSO_4_] × 10^2^ (mol kg^−1^)	Density (*d*) (g cm^−3^)				
	303.15 K	308.15 K	313.15 K	318.15 K	323.15 K
**Water**					
1.0	0.99714	0.99535	0.99340	0.99127	0.98898
3.0	1.00038	0.99830	0.99611	0.99375	0.99125
5.0	1.00407	1.00161	0.99911	0.99655	0.99390
7.0	1.00794	1.00533	1.00245	0.99955	0.99690
9.0	1.01225	1.00904	1.00615	1.00318	0.99975

**1.0% aqueous maltose**					
1.0	1.01861	1.01520	1.01309	1.01000	1.00788
3.0	1.02150	1.01795	1.01556	1.01233	1.01011
5.0	1.02480	1.02115	1.01850	1.01499	1.01264
7.0	1.02889	1.02517	1.02215	1.01843	1.01595
9.0	1.03332	1.02925	1.02653	1.02294	1.01988

**3.0% aqueous maltose**					
1.0	1.03582	1.03009	1.02716	1.02137	1.01596
3.0	1.03771	1.03181	1.02839	1.02246	1.01695
5.0	1.03979	1.03387	1.03010	1.02406	1.01846
7.0	1.04252	1.03630	1.03251	1.02620	1.02041
9.0	1.04550	1.03891	1.03536	1.02895	1.02278

**5.0% aqueous maltose**					
1.0	1.05316	1.04810	1.04239	1.03313	1.02422
3.0	1.05377	1.04853	1.04273	1.03348	1.02433
5.0	1.05477	1.04943	1.04363	1.03403	1.02489
7.0	1.05607	1.05041	1.04465	1.03500	1.02591
9.0	1.05775	1.05242	1.04607	1.03680	1.02775

**1.0% aqueous lactose**					
1.0	1.03649	1.03414	1.03222	1.03033	1.02541
3.0	1.03687	1.03485	1.03314	1.03151	1.02666
5.0	1.03747	1.03593	1.03439	1.03314	1.02859
7.0	1.03942	1.03742	1.03633	1.03519	1.03095
9.0	1.04102	1.03942	1.03811	1.03749	1.03343

**3.0% aqueous lactose**					
1.0	1.04252	1.04074	1.03851	1.03719	1.03467
3.0	1.04343	1.04163	1.03974	1.03863	1.03629
5.0	1.04483	1.04339	1.04151	1.04073	1.03868
7.0	1.04662	1.04511	1.04363	1.04304	1.04125
9.0	1.04876	1.04749	1.04597	1.04538	1.04403

**5.0% aqueous lactose**					
1.0	1.06044	1.05557	1.04996	1.04376	1.04003
3.0	1.06176	1.05717	1.05180	1.04576	1.04218
5.0	1.06349	1.05939	1.05428	1.04843	1.04511
7.0	1.06565	1.06180	1.05673	1.05122	1.04818
9.0	1.06811	1.06457	1.05992	1.05477	1.05209

a The uncertainty of density data of copper(ii) sulfate solutions is in the range of 2.0–2.1 × 10^−3^ g cm^−3^.

**Table 4:** Three-dimensional variables (*A*_*i*_) and correlation coefficients (*R*) of the polynomial relationship between density and temperature^a^

[CuSO_4_] × 10^2^ (mol kg^−1^)	*A*_0_	*A*_1_ × 10^4^	*A*_2_ × 10^6^	*R*
**Water**				
1.0	1.0043	−1.3829	−3.3710	0.9999
3.0	1.0097	−2.2534	−2.8860	0.9999
5.0	1.0174	−4.0742	−1.2570	0.9999
7.0	1.0243	−5.3434	−0.2860	0.9998
9.0	1.0285	−4.9834	−1.4860	0.9997

**1.0% aqueous maltose**				
1.0	1.0414	−8.9891	4.5700	0.9981
3.0	1.0463	−9.8400	5.2000	0.9987
5.0	1.0505	−10.0731	4.9700	0.9988
7.0	1.0561	−10.5926	5.0900	0.9991
9.0	1.0580	−9.2666	3.2900	0.9988

**3.0% aqueous maltose**				
1.0	1.0550	−4.6137	−6.3430	0.9964
3.0	1.0605	−6.2197	−4.9430	0.9975
5.0	1.0640	−6.7683	−4.6570	0.9982
7.0	1.0677	−7.0697	−4.7430	0.9978
9.0	1.0697	−6.4629	−5.7710	0.9969

**5.0% aqueous maltose**				
1.0	1.0487	11.1443	−32.1430	0.9993
3.0	1.0498	10.9740	−32.2000	0.9994
5.0	1.0510	11.0251	−32.5710	0.9992
7.0	1.0554	9.4254	−30.7140	0.9991
9.0	1.0588	8.5560	−29.6000	0.9996

**1.0% aqueous lactose**				
1.0	1.0299	6.4860	−14.6000	0.9908
3.0	1.0269	8.0023	−15.9400	0.9884
5.0	1.0250	8.9871	−16.3700	0.9857
7.0	1.0311	6.5203	−12.9400	0.9788
9.0	1.0328	6.2466	−12.0900	0.9735

**3.0% aqueous lactose**				
1.0	1.0516	−2.5471	−1.6290	0.9970
3.0	1.0524	−2.7703	−0.8571	0.9960
5.0	1.0533	−2.7177	−0.3430	0.9937
7.0	1.0556	−3.3163	0.9430	0.9917
9.0	1.0590	−4.0740	2.2000	0.9945

**5.0% aqueous lactose**				
1.0	1.0995	−14.3889	4.8290	0.9979
3.0	1.0982	−13.1997	3.8570	0.9977
5.0	1.0595	−11.4183	2.3430	0.9970
7.0	1.0984	−11.8011	3.3710	0.9966
9.0	1.0988	−11.1566	3.8460	0.9961

a The uncertainty of the tabulated data of three-dimensional variables is ±0.2%.

**Table 5:** Apparent molar volume of copper(ii) sulfate solutions at different temperatures

[CuSO_4_] × 10^2^ (mol kg^−1^)	Apparent molar volume (*V*_ϕ_) (cm^3^ mol^−1^)				
	303.15 K	308.15 K	313.15 K	318.15 K	323.15 K
**Water**					
1.0	100.316 ± 0.706	117.433 ± 0.708	131.623 ± 0.709	143.888 ± 0.710	152.264 ± 0.712
3.0	91.290 ± 0.568	106.673 ± 0.569	119.461 ± 0.571	131.334 ± 0.572	142.633 ± 0.573
5.0	80.218 ± 0.539	97.013 ± 0.540	110.898 ± 0.542	122.047 ± 0.543	131.866 ± 0.544
7.0	72.764 ± 0.525	86.819 ± 0.527	102.141 ± 0.528	114.984 ± 0.530	121.955 ± 0.531
9.0	63.650 ± 0.517	81.167 ± 0.518	93.116 ± 0.520	103.814 ± 0.521	118.023 ± 0.523

**1.0% aqueous maltose**					
1.0	118.699 ± 0.691	129.370 ± 0.694	140.138 ± 0.695	149.081 ± 0.697	161.023 ± 0.699
3.0	109.702 ± 0.556	117.718 ± 0.558	130.392 ± 0.560	137.969 ± 0.561	145.265 ± 0.563
5.0	99.757 ± 0.528	106.404 ± 0.530	119.020 ± 0.531	129.015 ± 0.533	135.946 ± 0.534
7.0	84.484 ± 0.515	90.045 ± 0.516	104.094 ± 0.518	114.065 ± 0.520	120.785 ± 0.521
9.0	72.298 ± 0.506	80.228 ± 0.508	87.820 ± 0.509	94.016 ± 0.511	105.467 ± 0.513

**3.0% aqueous maltose**					
1.0	173.893 ± 0.680	186.751 ± 0.684	199.459 ± 0.686	208.975 ± 0.689	220.562 ± 0.693
3.0	159.611 ± 0.548	169.495 ± 0.551	189.434 ± 0.553	197.577 ± 0.556	205.180 ± 0.559
5.0	152.974 ± 0.520	159.386 ± 0.523	178.088 ± 0.525	185.281 ± 0.528	191.793 ± 0.531
7.0	141.268 ± 0.508	149.880 ± 0.511	163.522 ± 0.513	172.405 ± 0.516	179.765 ± 0.519
9.0	132.035 ± 0.500	142.573 ± 0.503	150.634 ± 0.505	158.573 ± 0.508	168.393 ± 0.511

**5.0% aqueous maltose**					
1.0	222.649 ± 0.669	229.117 ± 0.672	235.845 ± 0.676	241.673 ± 0.682	243.776 ± 0.688
3.0	213.809 ± 0.539	222.047 ± 0.542	227.794 ± 0.545	230.665 ± 0.550	240.255 ± 0.555
5.0	204.843 ± 0.513	211.915 ± 0.516	215.709 ± 0.518	224.614 ± 0.523	230.851 ± 0.528
7.0	196.985 ± 0.501	206.423 ± 0.504	208.833 ± 0.507	216.254 ± 0.512	220.398 ± 0.516
9.0	188.663 ± 0.494	192.716 ± 0.497	200.776 ± 0.500	202.749 ± 0.504	205.678 ± 0.509

**1.0% aqueous lactose**					
1.0	232.512 ± 0.679	226.223 ± 0.548	220.760 ± 0.522	200.162 ± 0.509	192.263 ± 0.502
3.0	228.345 ± 0.681	214.793 ± 0.549	204.984 ± 0.522	195.128 ± 0.510	184.178 ± 0.503
5.0	222.173 ± 0.682	206.343 ± 0.550	196.789 ± 0.523	183.223 ± 0.511	177.250 ± 0.504
7.0	211.234 ± 0.683	194.678 ± 0.551	182.656 ± 0.524	171.656 ± 0.511	162.779 ± 0.504
9.0	204.484 ± 0.687	190.614 ± 0.553	174.638 ± 0.526	161.747 ± 0.514	153.177 ± 0.506

**3.0% aqueous lactose**					
1.0	217.406 ± 0.675	209.431 ± 0.677	204.248 ± 0.678	195.157 ± 0.679	187.104 ± 0.681
3.0	206.813 ± 0.545	201.868 ± 0.546	189.799 ± 0.547	180.646 ± 0.547	172.816 ± 0.548
5.0	195.286 ± 0.518	184.209 ± 0.519	176.836 ± 0.519	165.205 ± 0.520	155.101 ± 0.521
7.0	183.180 ± 0.506	177.022 ± 0.507	166.455 ± 0.507	155.617 ± 0.508	144.927 ± 0.508
9.0	173.235 ± 0.499	165.557 ± 0.499	158.277 ± 0.500	149.850 ± 0.500	136.538 ± 0.501

**5.0% aqueous lactose**					
1.0	198.086 ± 0.664	186.250 ± 0.667	179.710 ± 0.671	169.400 ± 0.675	158.645 ± 0.677
3.0	183.623 ± 0.535	171.623 ± 0.537	162.482 ± 0.540	154.407 ± 0.543	146.314 ± 0.545
5.0	173.212 ± 0.509	157.305 ± 0.511	147.155 ± 0.513	138.834 ± 0.516	129.145 ± 0.518
7.0	163.098 ± 0.497	148.557 ± 0.499	140.810 ± 0.501	130.413 ± 0.504	119.770 ± 0.505
9.0	154.367 ± 0.490	139.959 ± 0.491	129.668 ± 0.493	117.840 ± 0.496	105.808 ± 0.497

Limiting apparent molar volume of copper(ii) sulfate solutions at different temperatures

**Table:** 

Concentration of solvent (w/v%)	Limiting apparent molar volume (*V*^0^_ϕ_) (cm^3^ mol^−1^)				
	Masson's equation				
	303.15 K	308.15 K	313.15 K	318.15 K	323.15 K
**Water**					
0	120.613 ± 0.296	137.311 ± 0.336	151.678 ± 0.372	164.342 ± 0.403	176.212 ± 0.432

**Aqueous maltose**					
1.0%	146.188 ± 0.856	157.818 ± 0.857	170.738 ± 0.836	180.323 ± 0.981	190.836 ± 0.814
3.0%	195.432 ± 0.708	208.381 ± 0.714	227.921 ± 0.720	237.495 ± 0.720	248.460 ± 0.723
5.0%	241.324 ± 0.637	249.401 ± 0.640	255.641 ± 0.646	261.939 ± 0.652	267.511 ± 0.656

**Aqueous lactose**					
1.0%	258.315 ± 0.665	251.453 ± 0.676	245.703 ± 0.689	236.161 ± 0.709	233.089 ± 0.734
3.0%	242.481 ± 0.680	235.194 ± 0.688	228.601 ± 0.701	219.284 ± 0.713	214.713 ± 0.745
5.0%	221.783 ± 0.683	210.503 ± 0.708	204.578 ± 0.733	196.534 ± 0.758	190.154 ± 0.793

**Table:** 

Concentration of solvent (w/v%)	Limiting apparent molar volume (*V*^0^_ϕ_) (cm^3^ mol^−1^)				
	Redlich, Rosenfeld, & Meyer equation				
	303.15 K	308.15 K	313.15 K	318.15 K	323.15 K
**Water**					
0	104.436 ± 0.256	120.734 ± 0.296	134.839 ± 0.330	147.136 ± 0.360	158.226 ± 0.388

**Aqueous maltose**					
1.0%	126.317 ± 0.740	136.059 ± 0.739	148.833 ± 0.729	158.136 ± 0.860	167.383 ± 0.714
3.0%	177.295 ± 0.643	188.426 ± 0.646	206.925 ± 0.654	215.854 ± 0.655	225.364 ± 0.656
5.0%	226.413 ± 0.597	234.366 ± 0.601	239.874 ± 0.603	246.054 ± 0.610	251.992 ± 0.617

**Aqueous lactose**					
1.0%	240.848 ± 0.620	232.301 ± 0.624	225.205 ± 0.632	214.382 ± 0.643	209.590 ± 0.660
3.0%	223.002 ± 0.626	215.583 ± 0.631	207.752 ± 0.637	198.003 ± 0.644	191.340 ± 0.664
5.0%	201.917 ± 0.622	189.467 ± 0.638	182.211 ± 0.653	173.755 ± 0.670	166.076 ± 0.693

The slope of Masson's equation 
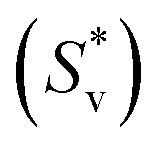
 and Redlich, Rosenfeld, & Meyer's equation (*b*_v_) at different temperatures

**Table:** 

Concentration of solvent (w/v%)	Slope 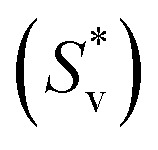 (cm^3^ kg^1/2^ mol^−3/2^)				
	303.15 K	308.15 K	313.15 K	318.15 K	323.15 K
**Water**					
0	−183.560 ± 0.450	−186.030 ± 0.456	−189.516 ± 0.464	−193.749 ± 0.475	−198.153 ± 0.485

**Aqueous maltose**					
1.0%	−231.774 ± 1.357	−249.980 ± 1.357	−256.483 ± 1.256	−261.421 ± 1.422	−269.172 ± 1.148
3.0%	−204.809 ± 0.742	−220.298 ± 0.755	−243.520 ± 0.769	−249.358 ± 0.756	−260.609 ± 0.759
5.0%	−169.282 ± 0.447	−174.098 ± 0.447	−178.302 ± 0.448	−182.537 ± 0.452	−185.227 ± 0.454

**Aqueous lactose**					
1.0%	−206.952 ± 0.532	−216.543 ± 0.582	−228.696 ± 0.641	−242.892 ± 0.729	−264.546 ± 0.833
3.0%	−222.805 ± 0.625	−224.125 ± 0.656	−233.084 ± 0.715	−235.492 ± 0.766	−261.054 ± 0.906
5.0%	−222.009 ± 0.684	−234.429 ± 0.789	−247.852 ± 0.888	−256.058 ± 0.988	−272.508 ± 1.136

**Table:** 

Concentration of solvent (w/v%)	Slope (*b*_v_) (cm^3^ kg mol^−2^)				
	303.15 K	308.15 K	313.15 K	318.15 K	323.15 K
**Water**					
0	−464.109 ± 1.137	−466.983 ± 1.144	−476.969 ± 1.169	−488.046 ± 1.196	−491.630 ± 1.204

**Aqueous maltose**					
1.0%	−594.920 ± 3.484	−634.833 ± 3.447	−659.962 ± 3.233	−675.724 ± 3.674	−683.791 ± 2.916
3.0%	−515.110 ± 1.867	−544.905 ± 1.867	−623.101 ± 1.968	−635.434 ± 1.927	−654.587 ± 1.906
5.0%	−428.796 ± 1.131	−447.182 ± 1.147	−450.785 ± 1.147	−466.856 ± 1.157	−486.097 ± 1.191

**Aqueous lactose**					
1.0%	−537.611 ± 1.383	−545.043 ± 1.464	−570.126 ± 1.599	−605.217 ± 1.817	−663.237 ± 2.090
3.0%	−564.689 ± 1.585	−568.027 ± 1.661	−581.723 ± 1.785	−583.768 ± 1.898	−650.933 ± 2.259
5.0%	−553.551 ± 1.705	−583.284 ± 1.963	−614.068 ± 2.199	−641.130 ± 2.473	−685.461 ± 2.858

**Table 8:** Standard limiting apparent molar volume of transfer at different temperatures

Concentration of solvent (w/v%)	Δ_t_*V*^0^_ϕ_ (cm^3^ mol^−1^)				
	303.15 K	308.15 K	313.15 K	318.15 K	323.15 K
**Aqueous maltose**					
1.0	25.575	20.507	19.060	15.981	14.624
5.0	120.711	112.090	103.963	97.597	91.299

**Aqueous lactose**					
1.0	137.702	114.142	94.025	71.819	56.877
5.0	100.131	73.192	52.900	32.192	11.782

**Table 9:** The value of Hepler's Constant in different solvents

Mathematical relationship	(δ^2^*V*^0^_ϕ_/δ*T*^2^)_*P*_ (cm^6^ mol^−2^ K^−2^)						
	Water	1.0% aq. maltose	3.0% aq. maltose	5.0% aq. maltose	1.0% aq. lactose	3.0% aq. lactose	5.0% aq. lactose
Masson's equation	−0.0644	−0.0149	−0.0265	−0.0334	0.0505	0.0362	0.0653
Redlich, Rosenfeld, & Meyer equation	−0.0694	−0.0066	−0.0216	−0.0269	0.0501	0.0101	0.0636

**Table 10:** Coefficients of the polynomial equation of *V*^0^_ϕ_ and *T* in different solvents

Solvent	*A* (cm^3^ mol^−1^)	*B* (cm^3^ mol^−1^ K^−1^)	*C* (cm^3^ mol^−1^ K^−2^)	*R*
Water	−3896.6364	23.0907	−0.0325	0.9999
1.0% aqueous maltose	−2090.5547	12.2013	−0.0159	0.9995
5.0% aqueous maltose	−1538.1235	10.1595	−0.0141	0.9999
1.0% aqueous lactose	1717.4787	−8.0932	0.0108	0.9973
5.0% aqueous lactose	2839.3470	−15.2891	0.0129	0.9967

**Table 11:** Limiting apparent molar expansibilities and isobaric thermal expansion coefficients at different temperatures

Solvent	303.15 K	308.15 K	313.15 K	318.15 K	323.15 K
***E*** ^**0**^_**ϕ**_ **(cm** ^**3**^ **mol** ^**−1**^ **K** ^**−1**^ **)**					
Water	3.386	3.061	2.736	2.411	2.086
1.0% aqueous maltose	2.561	2.402	2.243	2.084	1.925
5.0% aqueous maltose	1.611	1.470	1.329	1.188	1.047
1.0% aqueous lactose	−1.545	−1.437	−1.329	−1.221	−1.113
5.0% aqueous lactose	−2.011	−1.792	−1.573	−1.354	−1.135

***α***_**p**_ **× 10** ^**3**^ **(K** ^**−1**^ **)**					
Water	28.073	22.292	18.038	14.670	11.838
1.0% aqueous maltose	17.519	15.221	13.138	11.558	10.088
5.0% aqueous maltose	6.674	5.893	5.197	4.534	3.913
1.0% aqueous lactose	−5.982	−5.715	−5.410	−5.171	−4.776
5.0% aqueous lactose	−9.068	−8.514	−7.690	−6.890	−5.970

### 3.1. Density

The densities of pure water, aqueous maltose, and aqueous lactose are presented in Table 2. The densities of water are in good agreement with the values reported in the literature.^39–41^ Densities of copper(ii) sulfate solutions having a concentration range from 1.0–9.0 × 10^−2^ mol kg^−1^ in water, aqueous lactose, and aqueous maltose at temperatures of 303.15, 308.15, 313.15, 318.15, and 323.15 K are tabulated in Table 3. The uncertainty in the density of solvents and copper(ii) sulfate solutions was evaluated from the instrumental uncertainties in the mass and volume measurements by applying eqn (1).

**Fig. 1:**
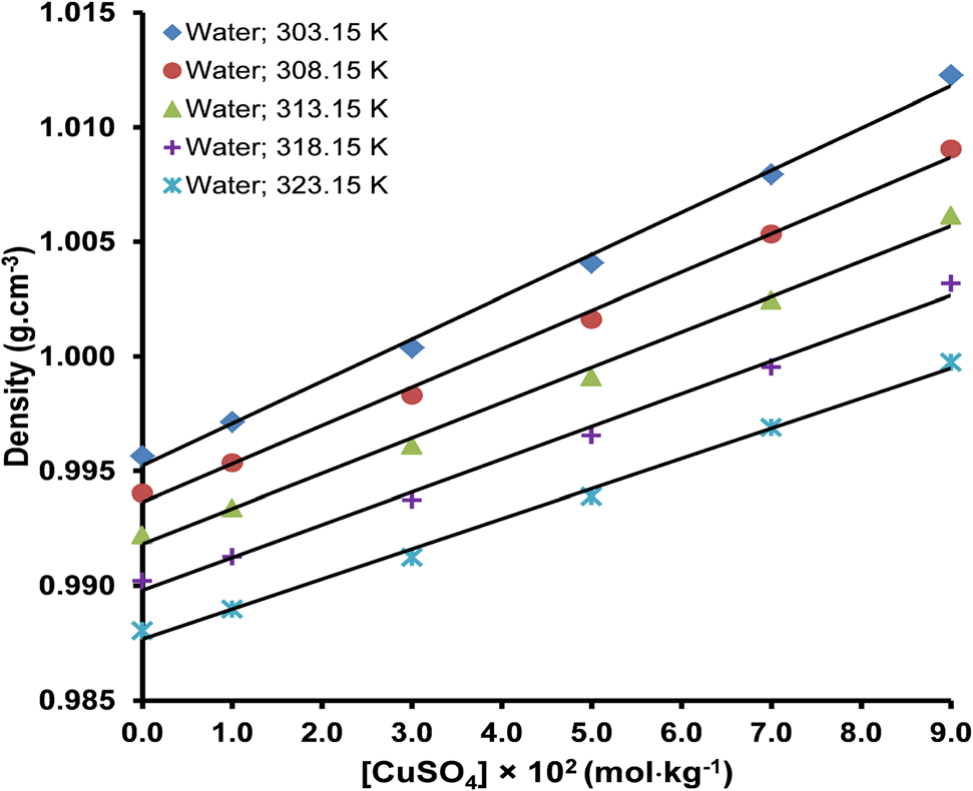
The effect of molality and temperature on the density of aqueous copper(ii) sulfate solutions (average error is ±0.20%).

**Fig. 2:**
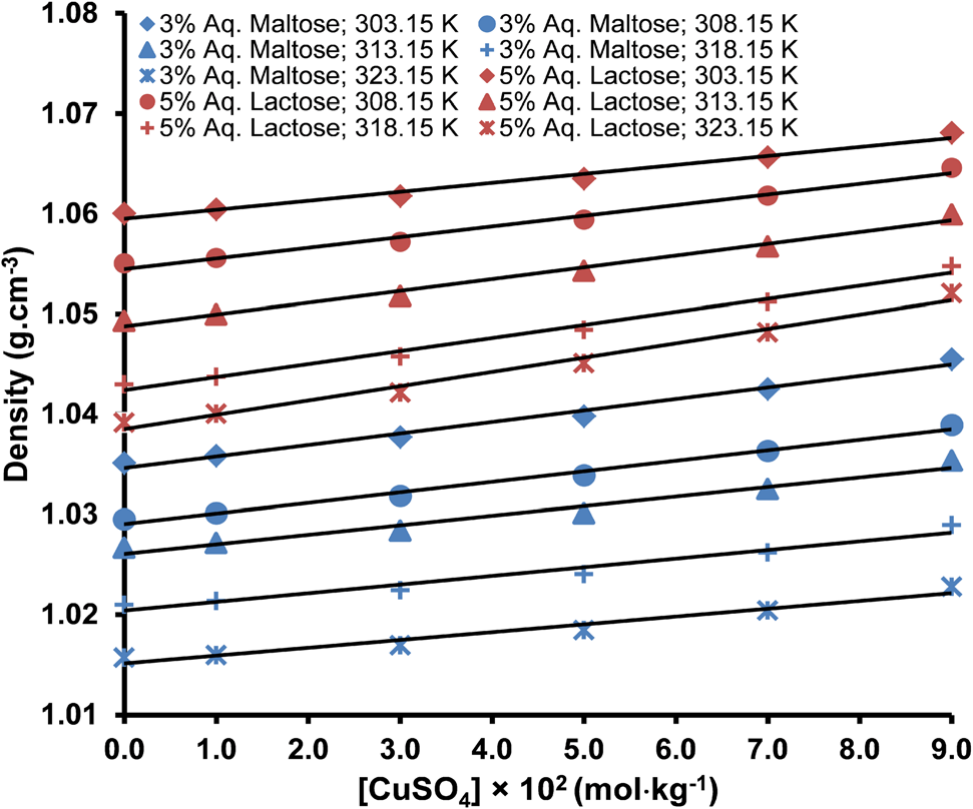
The effect of molality and temperature on the density of copper(ii) sulfate solutions in different solvents (average error is ±0.21%).

1 *U*_d_ = (*U*_m_/*m* + *U*_v_/*v*) × *d*

where *U*_m_, *U*_v_, and *U*_d_ represent the uncertainties in mass measurement, volume measurement, and density measurement, respectively. The density increased with an increase in the concentration of the copper(ii) sulfate (solute) and maltose/lactose (co-solute) in the water because both these materials are solids with a higher density as compared to water and produce strong intermolecular attractions in the solution due to their high charge.^42^ An increase in temperature decreases the density of the copper(ii) sulfate solutions because increased thermal agitation raises the kinetic energy of the ions and molecules in the solution, causing an expansion in the volume of the solution and a decrease in the ratio of mass per unit volume.^43^ The representative plots showing the effect of the nature of the solvent, concentration of copper(ii) sulfate, and temperature on the density of solutions are shown in Fig. 1 and 2.

The linear plots of density *versus* molality of solution in Fig. 1 and 2 are indicative of a directly proportional relationship between the density of the solution and its molality, which is proof of solute–solvent interaction of significant magnitude. The relationship between the density of a solution and the solute–solvent interaction inside the solution can be explained in the context of the free volume present in the solution. Free volume is the unoccupied space between different molecules in the three-dimensional structure of the bulk solvent and permits molecular movement and molecular interactions. The free volume is very sensitive to the addition of highly charged solute ions and temperature changes. Water is a liquid, and its molecules are rather loosely packed as compared to the solid copper(ii) sulfate; therefore, as the concentration of the copper(ii) sulfate in the solution is increased, the free volume inside the solution decreases, while an increase in temperature produces a reverse effect. Upon an increase in the molality of the solution, the increased number of charged ions (Cu^2+^ and SO_4_^2−^) form a more closely packed ion-molecular network, which strengthens the ion–solvent interactions and decreases the volume of the solution.^26,44,45^ The increase in density due to the increase in the concentration of maltose and lactose is also due to the same effect because both these molecules have multiple polar sites and interact strongly with water through hydrogen bonding.^46^

The relationship between the density and temperature of the water is complex due to various contributing factors, such as thermal expansion with the increase in temperature, due to the weakening/breaking/re-forming of hydrogen bonds, accompanied by a change in the structure of water molecules, and three-dimensional packing of water molecules in the bulk liquid. At a constant molality and pressure, the density of the solution and its temperature can be related by a polynomial mathematical model presented in eqn (2).^47,48^

**Fig. 3:**
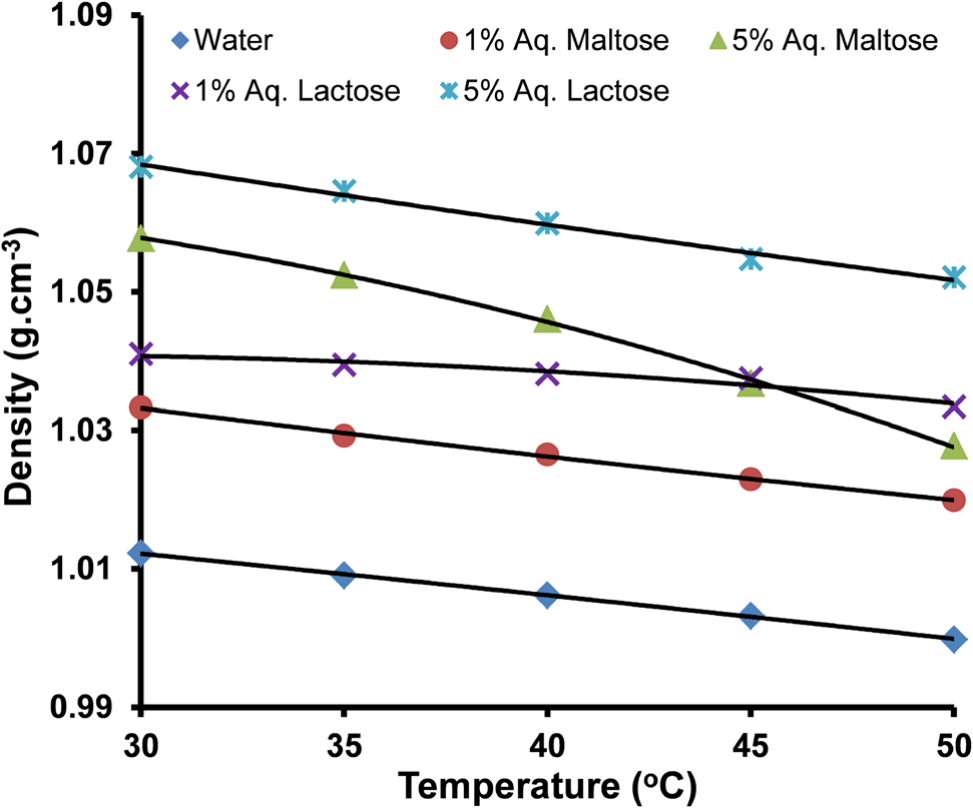
The polynomial relationship of density and temperature in different solvents (the coefficients of the polynomial equation of density *vs.* temperature and the correlation coefficients are presented in Table 4).

2 *d* = *A*_0_ + *A*_1_(*T* − 273.15) + *A*_2_(*T* − 273.15)^2^

where *A*_0_, *A*_1_, and *A*_2_ are the three-dimensional variables and *T* is the temperature in kelvin. The values of *A*_0_, *A*_1_, and *A*_2_ presented in Table 4 are obtained by the polynomial plots of density *versus* temperature (Fig. 3). The correlation coefficient (*R*) is in the range of 0.974–0.999, confirming the validity of the polynomial relationship between the density of copper(ii) sulfate solutions and temperature. This relationship between the density and temperature of water and other liquids is useful in predicting the temperature-dependent behavior and properties of solvents for engineering applications.^49^

### 3.2. Apparent molar volume (*V*_ϕ_)

The apparent molar volume (*V*_ϕ_) is the volume occupied by one mole of a solute in a solution.^50^ The apparent molar volume of the copper(ii) sulfate solution at different temperatures and solvent compositions was measured by using the density of the solvent and solution according to eqn (3).^51,52^

3 *V*_ϕ_ = *M*/*d* − 1000(*d* − *d*_o_)/*mdd*_o_

where *V*_ϕ_ is the apparent molar volume of solution in units of cm^3^. mol^−1^, *d* and *d*_o_ represent the density of solvent and solution, respectively, *M* is the molar mass of solute, and *m* is the molality of the solution. The possible sources of uncertainty in the value of apparent molar volume (*V*_ϕ_) are the uncertainties in the molality and density of the solution. The uncertainty in the molality of dilute solutions (≤0.10 mol kg^−1^) is typically very low (0.03%) and hence can be ignored in the uncertainty estimation of apparent molar volume (*V*_ϕ_). Therefore, the uncertainty in *V*_ϕ_ has been calculated by considering only the uncertainty in density measurement by using eqn (4).^53^

**Fig. 4:**
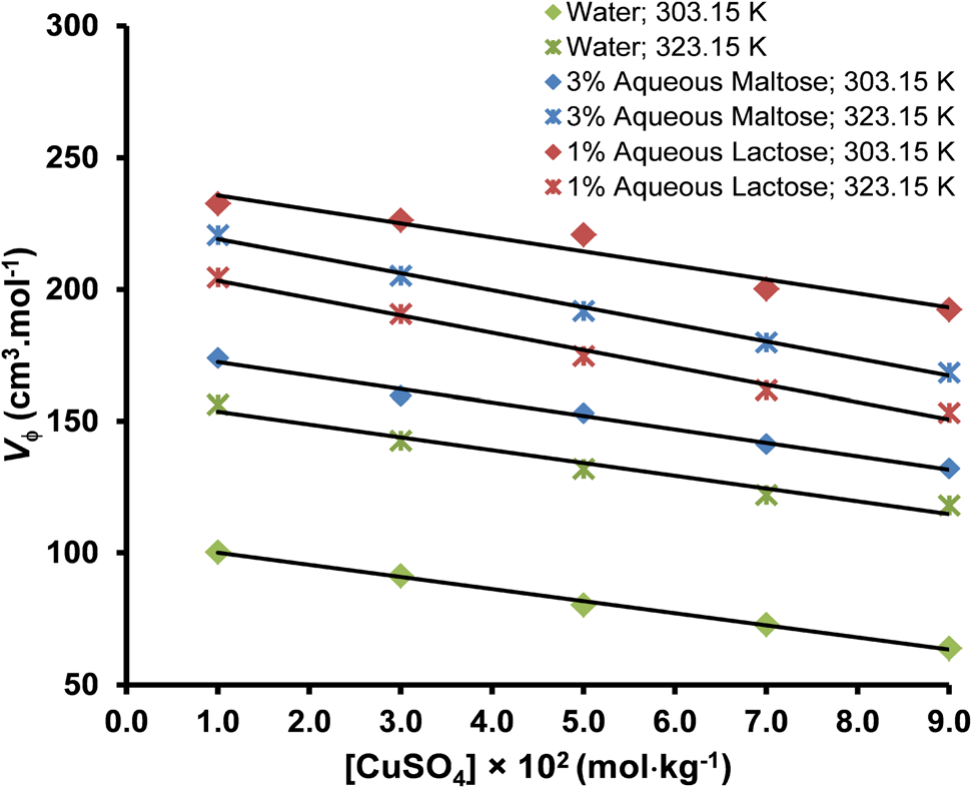
The effect of molality on the apparent molar volume of copper(ii) sulfate solutions at different temperatures.

4 *U*_*V*_ϕ__ = −(*M* + 1/*m*)(*U*_d_/*d*^2^)

where *U*_*V*_ϕ__ and *U*_d_ represent the uncertainties in the apparent molar volume and density, respectively. The values of apparent molar volume along with their standard uncertainties are tabulated in Table 5, whereas the effect of the molality of solution, temperature, and the concentration of solvent on the apparent molar volume is graphically illustrated in Fig. 4.

The apparent molar volume (*V*_ϕ_) is a measure of the volume occupied by the solute molecules in the solution and the volumetric changes in the solution due to the ionic interaction between the different components of the solution.^54^ The *V*_ϕ_ provides valuable information about the structure of solute, solute–solvent interactions, and thermodynamic properties of the solution. The magnitude of *V*_ϕ_ for a specific solute is usually less than its molar volume and is affected by the concentration of the solute and the temperature of the solution.^55^ The volumetric properties of an electrolyte solution are also affected by the formation of strongly associated ion pairs between highly charged ions like Cu^2+^ and SO_4_^2−^ due to a decrease in the number of free ions in the solution and decreasing its volume however, this phenomenon is not likely to occur at very low solute concentration of <0.1 mol kg^−1^ used in our study.

The value of *V*_ϕ_ is positive at all the experimental conditions, indicating that the solute ions are efficiently solvated due to the presence of strong solute–solvent interactions. The *V*_ϕ_ of copper(ii) sulfate solutions decreases with the increase in the concentration of solute in the solution due to the strong interaction between solute ions and solvent molecules, leading to a compression in the volume of the solution.^56^ The *V*_ϕ_ increases with the increase in the experimental temperature in water and aqueous maltose solvent due to the increased thermal agitation of molecules, leading to the weakening of hydrogen bonds and breaking of water molecules from the bulk water structure however, a decrement is observed in the aqueous lactose solvent.^57,58^ An increase in the concentration of maltose in the solution increased the *V*_ϕ_ while an increase in the concentration of lactose had the opposite effect.

In an aqueous solution, the net solution volume is affected by the electrostatic interactions between the solute ions and water molecules. Upon addition of a solute to the solvent, the added solute ions occupy the interstitial spaces between the solvent molecules, and the charge of the solute ions forces a rearrangement in its three-dimensional molecular arrangement. This structural rearrangement of molecules in the solvent may either decrease or increase the volume of the solution, depending on the concentration of solute and the nature of the interaction between the solute ions and solvent molecules.^59^ Similarly, a rise in temperature increases the collisional frequency and the interaction of the solute ions and solvent molecules, causing a contraction in the volume of the solution, while at the same time, thermal expansion also occurs due to the distortion of the bulk solvent structure as a result of the weakening of the intermolecular forces. In our study, the relative decrease in the density of copper(ii) sulfate solutions with the rise in temperature in water and aqueous maltose is greater than the respective pure solvents because the thermal expansion has outweighed the contraction in the volume of the solution due to the distortion in the bulk solvent structure caused by the increased solute–solvent interactions. However, in aqueous lactose, one glucose and one galactose molecules are linked through an β-1,4-glycosidic bond giving it an extended structure, and exposed hydroxyl (OH) groups that can interact with the water molecules and solute ions (Cu^2+^ and SO_4_^2−^) through hydrogen bonding resulting in a relatively less decrease in the density of the copper(ii) sulfate solution in aqueous lactose as compared to the aqueous maltose solvent. Hence, a decrease in the relative increase in density with the increasing concentration of copper(ii) sulfate is observed at higher temperatures, resulting in a decrease in the apparent molar volume of the solution.^60^

### 3.3. Limiting apparent molar volume and ionic interactions

**Fig. 5:**
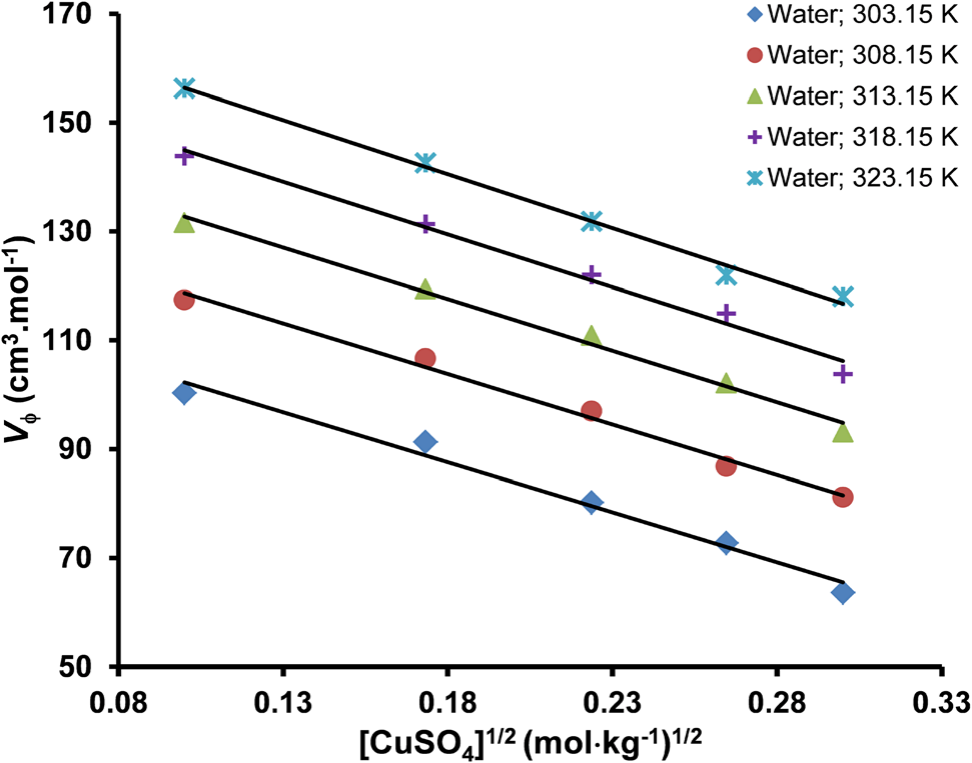
A straight-line plot of Masson's equation for the aqueous copper(ii) sulfate solutions at different temperatures (*R*: 0.992–0.996).

**Fig. 6:**
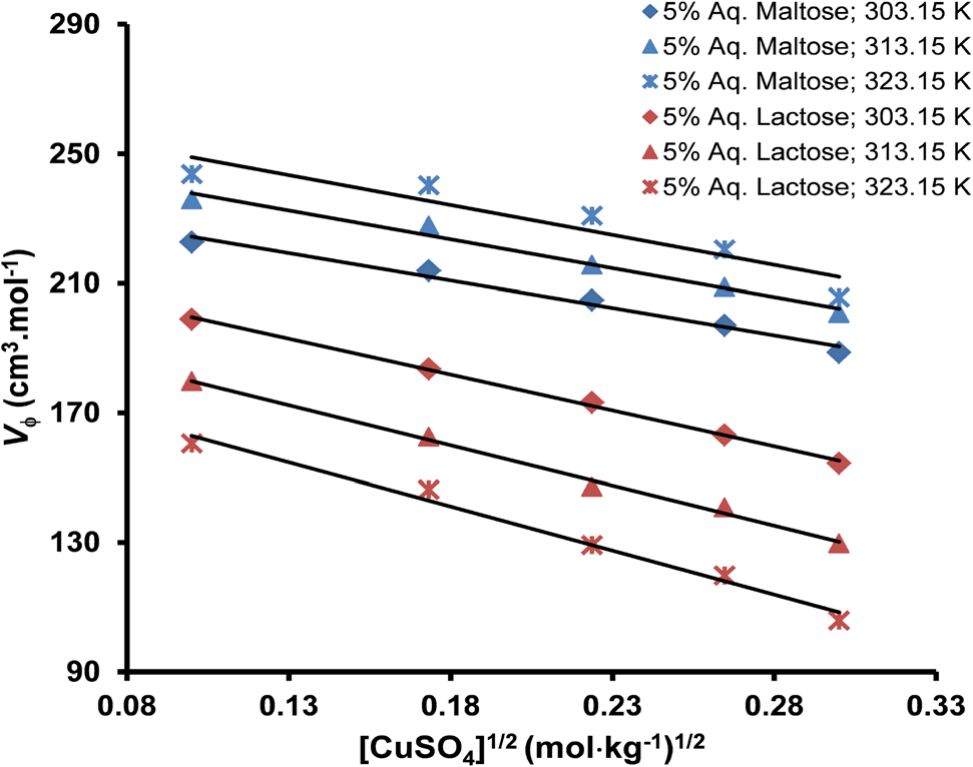
A straight-line plot of Masson's equation for the copper(ii) sulfate solutions in aqueous maltose and aqueous lactose solvents at different temperatures (*R*: 0.938–0.999).

Limiting apparent molar volume (*V*^0^_ϕ_) can be evaluated by using Masson's equation (eqn (5)) based on the straight-line relationship of the apparent molar volume (*V*_ϕ_) and the square root of molality.^51,61,62^5
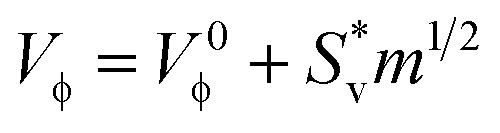
where *V*^0^_ϕ_ is the limiting apparent molar volume of the copper(ii) sulfate solutions and 
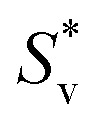
 is the slope of Masson's equation. The plots of Masson's equation were linear with a good correlation coefficient (*R*) in the range of 0.938–0.999, confirming that Masson's equation is perfectly applicable to the density data of the copper(ii) sulfate solutions and indicating a strong positive relationship between the *V*_ϕ_ and *m*^1/2^. The plots of Masson's equation are presented in Fig. 5 and 6, while the values of intercept (*V*^0^_ϕ_) and slope 
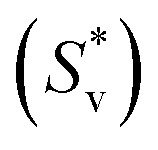
 are presented in Tables 6 and 7, respectively.

The relationship between the apparent molar volume and molality of the solution in the dilute region can also be evaluated by the Redlich, Rosenfeld, & Meyer relationship shown in eqn (6).^63,64^

6 *V*_ϕ_ = *V*^0^_ϕ_ + *A*_V_*m*^1/2^ + *b*_v_*m*

where *b*_V_ is an empirical parameter and *A*_V_ is the Pitzer–Debye–Huckel limiting slope for apparent molar volume. The values of the Pitzer–Debye–Huckel limiting slope for water at different temperatures have been reported earlier.^65^Eqn (6) can be rearranged to evaluate the graphical correlation of the apparent molar volume and molality of the solution as shown in eqn (7).

7 *V*_ϕ_ − *A*_V_*m*^1/2^ = *V*^0^_ϕ_ + *b*_v_*m*

**Fig. 7:**
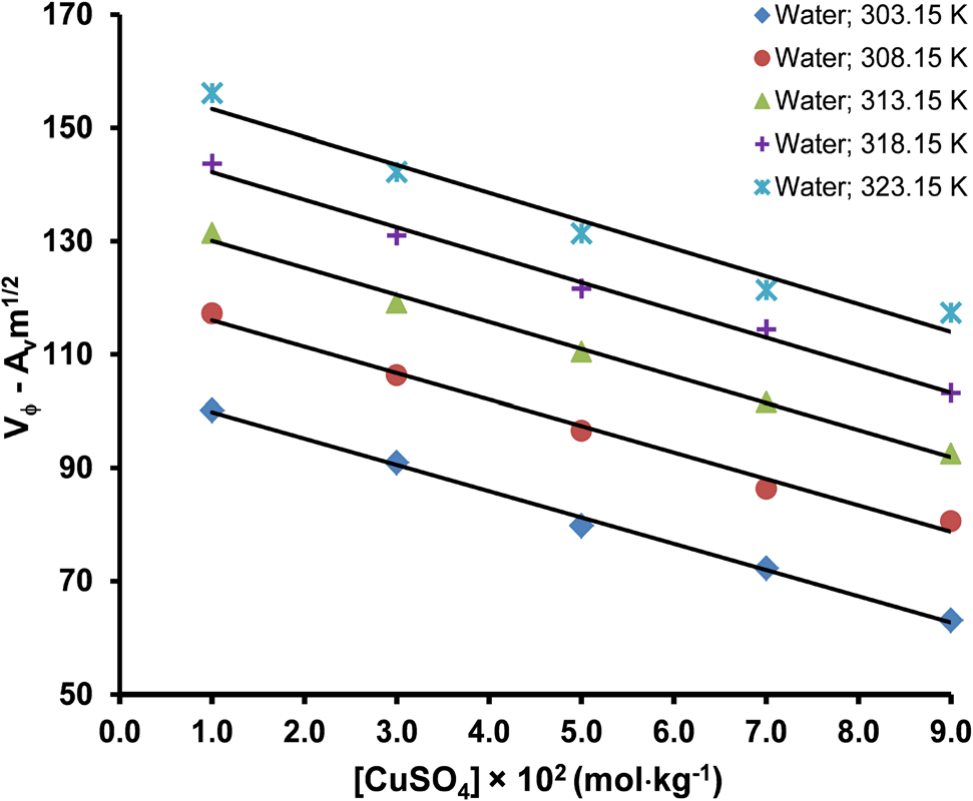
A straight-line plot of the Redlich, Rosenfeld, & Meyer equation for the aqueous copper(ii) sulfate solutions at different temperatures (*R*: 0.992–0.997).

**Fig. 8:**
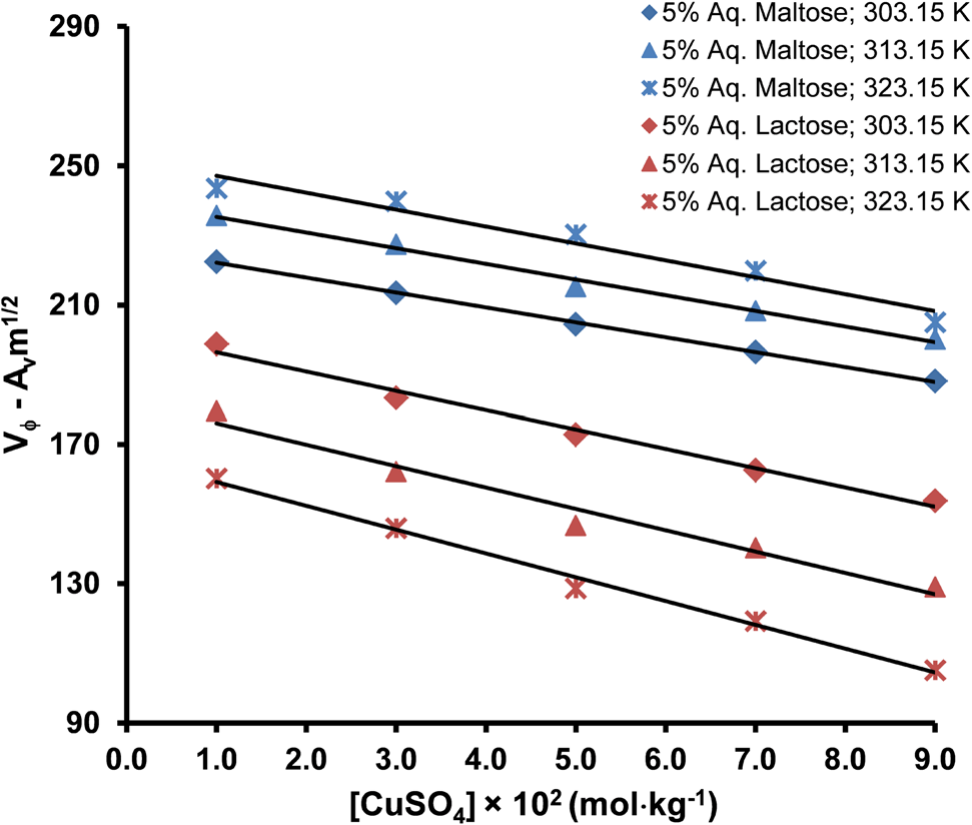
A straight-line plot of the Redlich, Rosenfeld, & Meyer equation for the copper(ii) sulfate solutions in aqueous maltose and aqueous lactose solvents at different temperatures (*R*: 0.939–0.999).

The plots of the Redlich, Rosenfeld, & Meyer equation (Fig. 7 and 8) were linear with high correlation coefficient (*R*) values in the range of 0.973–0.998, confirming its applicability to the experimental data. The values of intercept (*V*^0^_ϕ_) and slope (*b*_v_) are presented in Tables 6 and 7, respectively. In Masson's and Redlich, Rosenfeld, & Meyer's equation, the *V*^0^_ϕ_ represents the solute–solvent interactions, whereas 
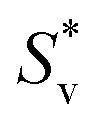
 and *b*_v_ provide a measure of the solute–solute or ion–ion interactions. A positive value of *V*^0^_ϕ_ and a negative value of 
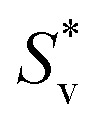
 and *b*_v_ indicate that the solute–solvent interactions are stronger than the solute–solute interactions.^66^ A comparative analysis of the values of limiting apparent molar volume (*V*^0^_ϕ_) calculated by Masson's equation and the Redlich, Rosenfeld, & Meyer equation presented in Table 6 shows that the magnitude of *V*^0^_ϕ_ obtained by Masson's equation is relatively higher as compared to the values calculated by the Redlich, Rosenfeld, & Meyer equation however the *V*^0^_ϕ_ values obtained by these two equations follow similar trends concerning the temperature and concentration of solvent. Hence, either one or both of these relationships can be used to evaluate the nature of ionic interaction inside the copper(ii) sulfate solution in water, aqueous maltose, and aqueous lactose solvents.

Limiting apparent molar volume (*V*^0^_ϕ_) of a solution is a thermodynamic property and represents the value of apparent molar volume at effectively zero concentration of solute. In such a condition, the ion–ion interaction is practically zero because the ions are present at very large distances and are only surrounded by the solvent molecules. Hence, *V*^0^_ϕ_ provides a very good approximation of the solute–solvent interactions in a solution and the associated volumetric changes due to the formation of a solvation shell around the solute ions.^67^ The value of *V*^0^_ϕ_ is affected by the charge of the electrolyte ions, the hydration number of solute ions, and the molar composition of the solution.^68^

**Fig. 9:**
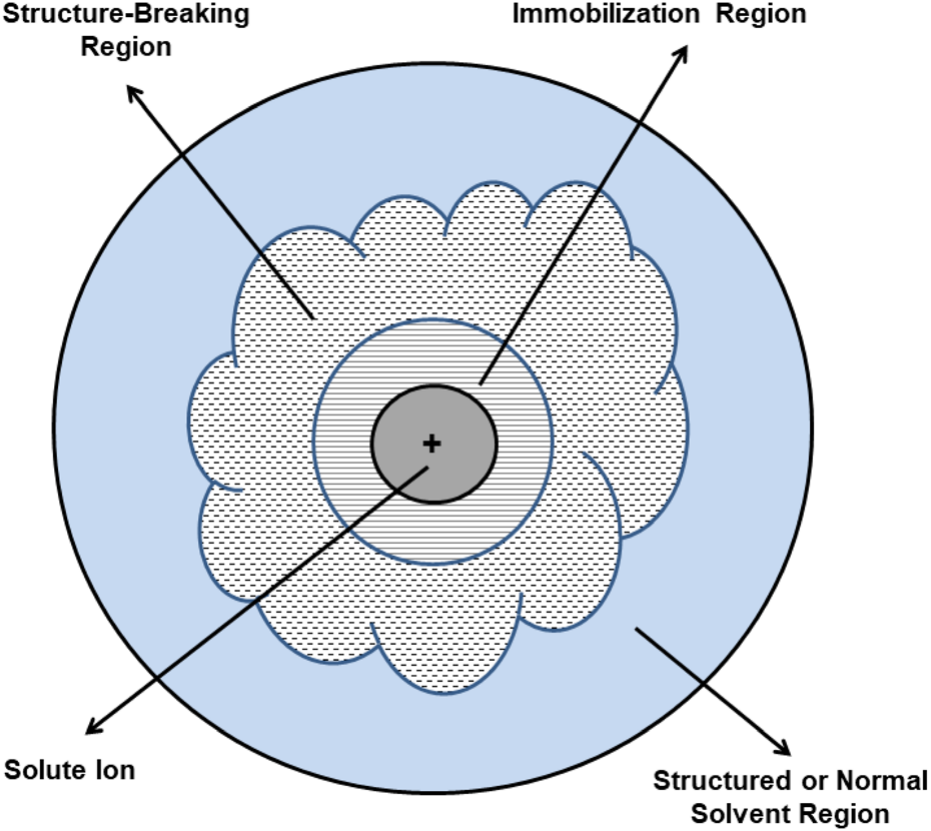
A model of the distortion in bulk solvent structure by the solute ions.

The values of limiting apparent molar volume (*V*^0^_ϕ_) of copper(ii) sulfate solutions in water obtained in our study are in good agreement with the values reported earlier.^69^ In the present study, the values of *V*^0^_ϕ_ in water increase with the addition of maltose and lactose in the solution because these molecules act as co-solute in the solution. The values of *V*^0^_ϕ_ are relatively larger in aqueous lactose solvent as compared to the aqueous maltose solvent due to the difference in the three-dimensional structure of the two different disaccharides and more exposed hydrogen bonding sites in lactose, thereby strongly interacting with the water molecules and producing a positive volume change by pulling out the water molecules from the solvation layer of solute ions. The *V*^0^_ϕ_ increased with a rise in temperature in aqueous maltose solvent, whereas it decreased with the increase in temperature in aqueous lactose solvent. An increase in *V*^0^_ϕ_ with temperature is due to the increased thermal agitation of molecules in the solution which weakens the electrostatic force of attraction between the water molecules in the bulk solvent so that the water molecules start breaking off from the bulk solvent and more monomeric water molecules are available to solvate the solute ions leading to an increase in the apparent molar volume (*V*^0^_ϕ_) of the solute.^59^ The decrease of *V*^0^_ϕ_ with an increase in temperature is a rather unusual behavior, but such a behavior has been reported in the literature for the aqueous solution of copper(ii) sulfate, organic acids, amino acids, drug molecules, and ionic liquids.^69–72^ The decrease of *V*^0^_ϕ_ with an increase in temperature is an indication of the ordering in the bulk solvent structure and the structure-making behavior of solute due to an expansion of the hydrogen bonding network inside the copper(ii) sulfate solution by the interaction of lactose molecules with water molecules and solute ions especially SO_4_^2−^ ions due to the presence of multiple hydrogen bonding sites. The variation in *V*^0^_ϕ_ with the change in solution temperature can be explained in the light of Frank and Wen's model, as shown in Fig. 9.^73,74^ This model assumes that the bulk aqueous solutions of electrolytes consist of three distinct regions:

(a) An immobilization region in which the water molecules are strongly attached to the solvated ions.

(b) A random intermediate or structure-breaking region where the inherent three-dimensional structure of water is perturbed.

(c) A structured region where the water exists in its normal state.

The increase in temperature triggers the movement of water molecules from the electrostricted solvation layer of solute ions to the normal region, thereby producing a positive volume change, whereas an increase in temperature also breaks the tetrahedral clustering of water molecules, producing a negative volume change. Upon increase of the solution temperature in the present study, the water molecules move from the solvation sheath of the solute ions to the bulk water producing an increase in the limiting apparent molar volume (*V*^0^_ϕ_) in aqueous maltose but in the case of aqueous lactose, the hydrogen bonding between the water and lactose molecules causes a contraction of the bulk water by disrupting its inherent tetrahedral clustering producing a decrease in the limiting apparent molar volume (*V*^0^_ϕ_). Hence, a continuous decrease in the *V*^0^_ϕ_ with the increase in temperature and the concentration of lactose in the solvent is observed.^75,76^ The ions with high charge, such as Cu^2+^ pull the water molecules from the structured region to the distorted region, whereas large ions like SO_4_^2−^ easily release water molecules from the distorted region in the near vicinity of the solute ion to the structured region, producing a positive volume change. The SO_4_^2−^ is a complex anion and also promotes hydrogen bonding in bulk water due to the presence of highly electronegative oxygen atoms.^77^ Hence, it can be concluded that SO_4_^2−^ contributes more to the apparent molar volume (*V*_ϕ_) as compared to the Cu^2+^ ions.

The value of the experimental slope 
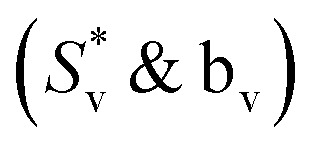
 provides an in-depth view of the magnitude of interactions between solute ions in a solution. A large negative value of 
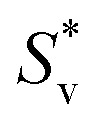
 and *b*_v_ obtained in our study is indicative of very weak solute–solute interactions due to the complete dissociation of the copper(ii) sulfate into its constituent ions, Cu^2+^ and SO_4_^2−^.^78,79^ The value of 
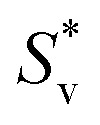
 & *b*_v_ is affected by the composition and temperature of the solvent because these parameters affect the solvation behavior of the solute. The value of 
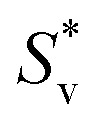
 & *b*_v_ decreases with the increase in temperature because an increase in the volume of the solution increases the inter-ionic distances and hence the ion–ion interaction decreases with the increase in temperature.

### 3.4. Limiting apparent molar volume of transfer (Δ_t_*V*^0^_ϕ_)

The standard apparent molar volume of transfer (Δ_t_*V*^0^_ϕ_) is the difference in the *V*^0^_ϕ_ of the solute when it is transferred from one solvent to another, such as from water to aqueous maltose or aqueous lactose. The Δ_t_*V*^0^_ϕ_ of copper(ii) sulfate solutions from water to the aqueous maltose/lactose is calculated by eqn (8).^80^

8 Δ_t_*V*^0^_ϕ_ = *V*^0^_ϕ_(aqueous maltose/lactose) − *V*^0^_ϕ_(water)

The values of Δ_t_*V*^0^_ϕ_ presented in Table 8 are positive and decrease with an increase in the temperature. The variation of *V*^0^_ϕ_ can be interpreted based on structural changes inside the solution in the light of the cosphere overlap model. This model assumes that the ions and molecules in solution are spheres and that when two such species interact with each other, the volume of the solution is affected depending upon the nature of the interacting species due to the displacement of some of the material from the cosphere, producing a change in the volumetric properties of the solution.^81–85^ Depending upon the nature of the interacting cospheres, there are three possibilities.

(a) If X and Y are both hydrophobic, Δ*V* < 0.

(b) If X is hydrophobic and Y is ionic or dipolar, Δ*V* < 0.

(c) If X and Y are both ionic or dipolar, Δ*V* > 0.

**Fig. 10:**
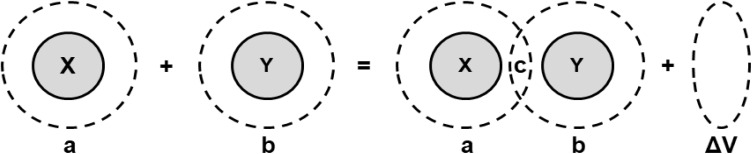
The structural interaction between two cospheres.^84,85^

A diagrammatic illustration of the interaction of cospheres is presented in Fig. 10.

In our study, copper(ii) sulfate is ionic, and maltose/lactose has multiple polar sites; therefore, positive values of the limiting apparent molar volume of transfer (Δ_t_*V*^0^_ϕ_) are obtained. The limiting apparent molar volume (*V*^0^_ϕ_) of a solution is the contribution of various other volumes as shown in eqn (9).^80,86^

9 *V*^0^_ϕ_ = *V*_vw_ + *V*_void_ − *nV*_shrinkage_

where *V*_vw_ is the van der Waals volume, *V*_void_ is the volume of vacant spaces present in the solution matrix, *V*_shrinkage_ is the decrement in the solvated volume of solute due to the interaction of co-solute molecules with the solvent, and *n* is the potential number of hydrogen bonding sites in the co-solute molecule. If we consider maltose/lactose as a co-solute, then an increase in the concentration of maltose/lactose would cause a slight increase in *V*_vw_ due to the presence of polar sites in the molecules but *V*_void_ and *V*_shrinkage_ would decrease because these co-solute molecules will fill some of the vacant spaces present in the solution matrix which will decrease the number of water molecules in the hydration sheath around the Cu^2+^ and SO_4_^2−^ ions. This decrease in the number of water molecules from the hydration layer of the solute ions reduces the electrostatic constraint on these ions, which decreases the *V*_shrinkage_ of the solute, and the *V*^0^_ϕ_ of the solution is increased.^87^ Hence, Δ_t_*V*^0^_ϕ_ values are positive and reflect the dehydration effect of maltose/lactose on the Cu^2+^ and SO_4_^2−^ ions. A molecular-level structural interpretation of the CuSO_4_–H_2_O–maltose/lactose system reveals that there can be four types of interactions.^87^

(a) Ion–dipole interactions between the Cu^2+^ ions and the oxygen atoms of water and maltose/lactose.

(b) Ion–dipole interactions between the SO_4_^2−^ ions and the hydrogen atoms of water and maltose/lactose.

(c) Hydrophilic–hydrophilic interactions between the hydroxyl and glycosidic groups of the polar water molecules and maltose/lactose, respectively.

(d) Hydrophobic–hydrophilic interactions between the –CH_2_ groups of maltose/lactose and the polar water molecules.

The cosphere overlap model states that a, b, and c types of interactions contribute positively, while the d type of interactions contributes negatively to the Δ_t_*V*^0^_ϕ_. Hence, it can be concluded that the positive values of Δ_t_*V*^0^_ϕ_ are due to the dominance of the interactions of types a, b & c because of the ionic and/or polar nature of the species and/or groups involved in the interaction.^88,89^

### 3.5. Structure-breaking/structure-making property of solute

The water molecule has sp^3^ hybridization and a tetrahedral structure, which makes it one of the most unique liquids in the universe.^90^ In water and aqueous solutions, the three-dimensional structure of the solution resembles a cage in which the water molecules occupy their fixed positions in the lattice whereas the solute ions are entrapped in the interstitial spaces present between the water molecules, and hence the structure-making and structure-breaking can be imagined as the structuring and breaking down of the cage-like three-dimensional arrangement of water molecules around the solute ions/molecules.^91^ Limiting apparent molar volume (*V*^0^_ϕ_) is a measure of the solute–solvent interaction, and the structure-making or structure-breaking ability of a particular solute in a specific solvent can be evaluated by studying the variation in the value of *V*^0^_ϕ_ with the change in solution temperature.^92^ However, the most applicable criterion based on the solution thermodynamics for the evaluation of long-range structure-making or the structure-breaking ability of solute concerning a specific solvent system is Hepler's constant (eqn (10)).^91,93–96^

10 (δ*C*_P_/δ*P*)_*T*_ = −*T*(δ^2^*V*^0^_ϕ_/δ*T*^2^)_*P*_

If the value of (δ*C*_P_/δ*P*)_*T*_ is negative or very small positive, the solute is hydrophilic and behaves as a structure-breaker, whereas a positive value is characteristic of a structure-making hydrophobic solute.^91^ The value of Hepler's constant for copper(ii) sulfate in water, aqueous maltose, and aqueous lactose solvents by utilizing the limiting apparent molar volume (*V*^0^_ϕ_) data obtained by Masson's and Redlich, Rosenfeld, & Meyer equations are presented in Table 9. The value of Hepler's constant in the water and different compositions of aqueous maltose solvent is negative, confirming the structure-breaking behavior of copper(ii) sulfate in the aqueous maltose solvent, whereas a positive or very small negative value in aqueous lactose solvent indicates that the copper(ii) sulfate behaves as a structure-maker in aqueous lactose solvent. Therefore, it can be concluded with confidence that the behavior of copper(ii) sulfate in terms of structure-making or structure-making ability is different in the two solvents used in the present study and that the copper(ii) sulfate behaves as a structure-breaker in the aqueous maltose solvent, whereas it acts as a structure-maker in the aqueous lactose solvent. The structure-breaking behavior of copper(ii) sulfate has also been reported earlier in water and aqueous dextrose, whereas it has been reported to behave as a structure-maker in an aqueous propylene glycol.^69,97^

The structure-breaking/promoting behavior of the solute can be rationalized in the light of the Flickering Cluster model.^98–100^ This model proposes that the bulk water consists of a highly organized molecular region and some free molecules. There is a continuous exchange of water molecules between these two regions, which results in the formation of two different long-lived structures inside the liquid. The bulk water can be viewed as a time-averaged structure in which the monomeric water molecules are in a dynamic equilibrium with the water clusters. Once a solute is added to the solvent, the solute ions/molecules get surrounded by a sheath of water molecules. In the case of hydrophobic solutes with low charge, the solute molecule breaks the water molecules from the bulk, thereby behaving as a structure-breaking solute, whereas in the case of the structure-making solutes, the nature of solute–solvent interaction acts in a way that the structural order of the water is increased, as often observed in the case of solutes with high charge.^96^

### 3.6. Thermodynamic parameters

The relationship between the limiting apparent molar volume (*V*^0^_ϕ_) and temperature is rather complex due to its dependence on the nature and concentration of the solution components, and cannot be explained by a simple linear relationship. The temperature dependence of *V*^0^_ϕ_ was evaluated by the graphical analysis using a second-order polynomial equation concerning temperature (eqn (11)), and the coefficients of the equation were evaluated.^59,101,102^

11 *V*^0^_ϕ_ = *A* + *BT* + *CT*^2^

**Fig. 11:**
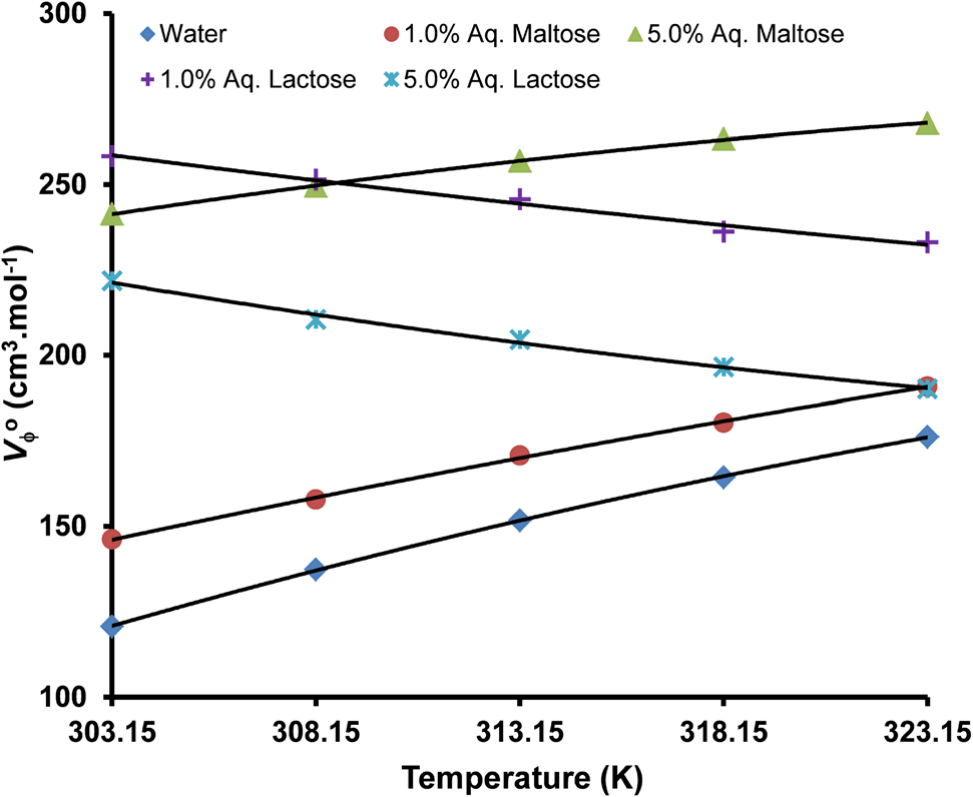
A plot of the polynomial relationship between limiting apparent molar volume (*V*^0^_ϕ_) and temperature (*T*) in different solvents (the coefficients of the polynomial equation and the correlation coefficients of *V*^0^_ϕ_*vs. T* are presented in Table 10).

A representative plot of the polynomial equation of *V*^0^_ϕ_ and temperature in water, aqueous maltose, and aqueous lactose solvents is presented in Fig. 11, while the values of its coefficients are tabulated in Table 10.

#### 3.6.1. Limiting apparent molar expansibility (*E*^0^_ϕ_)

Limiting apparent molar expansibility (*E*^0^_ϕ_) is the change in the apparent molar volume of a solute concerning temperature. The coefficients of eqn (11) were used to evaluate the limiting apparent molar expansibility (*E*^0^_ϕ_) of the copper(ii) sulfate solution by using eqn (12), and the values are presented in Table 11.^103,104^

12 *E*^0^_ϕ_ = (∂*V*^0^_ϕ_/∂*T*) = *B* + 2*CT*

The *E*^0^_ϕ_ is the sum of three contributive factors.^67,105^

13 *E*^0^_ϕ_ = *E*^0^_ϕ_(intrinsic) + *E*^0^_ϕ_(electrostatic) + *E*^0^_ϕ_(steric)

The *E*^0^_ϕ_(intrinsic) is the expansibility due to the void space around the ion, *E*^0^_ϕ_(electrostatic) is the expansivity due to a change in the hydration of solute whereas *E*^0^_ϕ_(steric) is the expansivity due to changes in the structure of bulk solvent due to the electrostriction of added ions.^67,105^ At low temperatures, the structural component *E*^0^_ϕ_(steric) is the major contributing factor to the magnitude of *E*^0^_ϕ_ whereas at higher temperatures, the *E*^0^_ϕ_(electrostatic) dominates.

**Fig. 12:**
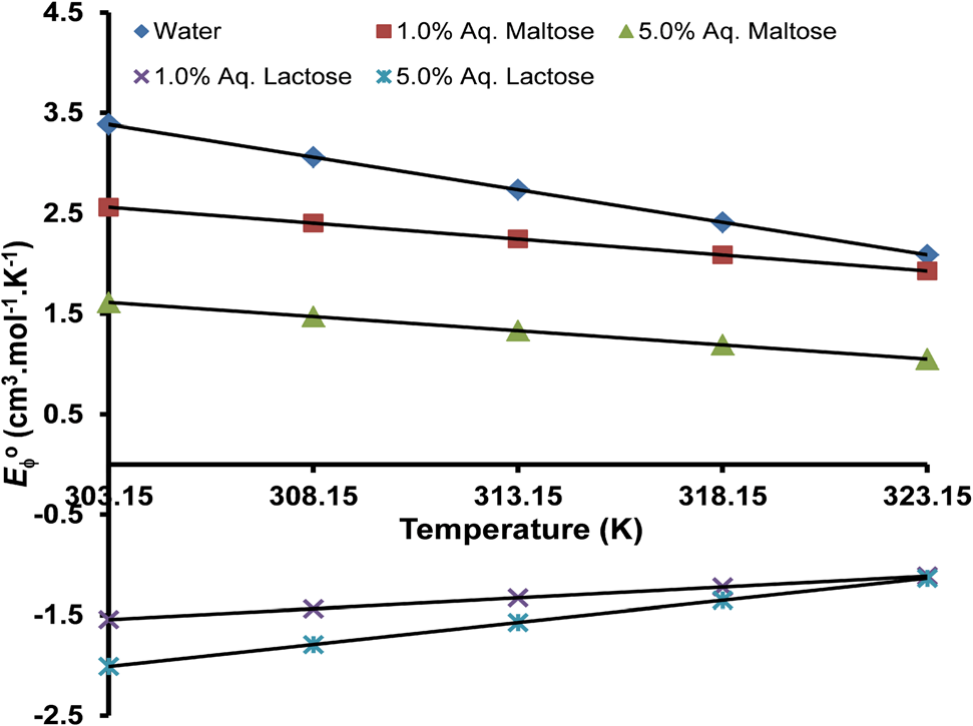
The variation of limiting apparent molar expansibility (*E*^0^_ϕ_) with the temperature (*T*) in different solvents.

The magnitude of *E*^0^_ϕ_ is related to the thermodynamic changes during the solvation of solute ions in the solution and therefore can be used as a marker for evaluating the strength of solute–solvent interactions. The value of *E*^0^_ϕ_ is strongly affected by the concentration of solvent and the change in experimental temperature. In water and aqueous maltose, the values of *E*^0^_ϕ_ are positive due to the presence of strong solute–solvent interactions in which each solute ion is surrounded by a large number of water molecules, and these values decrease with the rise in temperature per the structure-making behavior.^106^ The positive value of *E*^0^_ϕ_ is due to the increased solvation and electrostriction of the solvent molecules around the solute ions. As the temperature of the solution rises, the degree of hydration of solute ions decreases, resulting in the release of a few water molecules from the solvation shell of the solute to the bulk solvent. This decreased solvation causes the solution to expand a little more rapidly than the pure water, giving positive *E*^0^_ϕ_.^107^ The decrease of *E*^0^_ϕ_ with the increase in temperature in water and aqueous maltose is due to the gradual appearance of the “caging effect” resulting in a more compact packing of the solution components, per the behavior of a structure-breaking solute.^108^ The negative *E*^0^_ϕ_ in aqueous lactose solvent is due to a negative volume change with the temperature rise, per the behavior of a structure-making solute.^109^ A graphical analysis of the variation in the limiting apparent molar expansibility (*E*^0^_ϕ_) of the copper(ii) sulfate solution with temperature is presented in Fig. 12.

#### 3.6.2. Isobaric thermal expansion coefficient (*α*_p_)

The isobaric thermal expansion coefficient (*α*_p_) is the ratio of limiting apparent molar expansibility (*E*^0^_ϕ_) and limiting apparent molar volume (*V*^0^_ϕ_). The *α*_p_ is a thermodynamic property of a solution and provides valuable information about the changes in the solvent structure upon the addition of solute ions. The isobaric thermal expansion coefficient (*α*_p_) for the copper(ii) sulfate solution in water, aqueous maltose, and aqueous lactose solvents was calculated by using eqn (14), and the values are presented in Table 11.^110,111^

14 *α*_p =_*E*^0^_ϕ_/*V*^0^_ϕ_

As can be seen from eqn (14), *α*_p_ and *E*^0^_ϕ_ are directly proportional to each other; therefore, the values of *α*_p_ for the copper(ii) sulfate solution exhibit the same trend as that of *E*^0^_ϕ_ concerning the experimental temperature and the concentration of solvent. The *α*_p_ decreased with the rise of temperature in the water, aqueous maltose solvent, due to a decrease in *E*^0^_ϕ_ and an increase in *V*^0^_ϕ_, whereas an opposite trend is observed in the aqueous lactose solvent.

## 4. Conclusion

In the present study, the density, volumetric, and thermodynamic properties of copper(ii) sulfate solutions in water, aqueous maltose, and aqueous lactose solvents have been studied. The positive and significantly large value of limiting apparent molar volume (*V*^0^_ϕ_) and negative values of experimental slope 
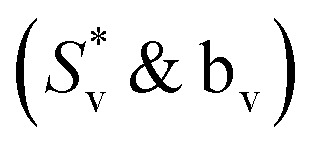
 obtained from the plots of Masson's equation and Redlich, Rosenfeld, & Meyer equation indicates that the solute–solvent interactions; copper(ii) sulfate-(water-maltose/lactose) are dominant over the solute–solute interactions and the same is confirmed by the positive values of the limiting apparent molar volume of transfer (Δ_t_*V*^0^_ϕ_). The negative values of Hepler's constant (δ^2^*V*^0^_ϕ_/δ*T*^2^)_*P*_ in water and aqueous maltose solvent confirmed that the copper(ii) sulfate behaves as a structure-breaker, while a positive value of Hepler's constant in aqueous lactose solvent confirmed that copper(ii) sulfate behaves as a structure-maker. A comparative analysis of the results and conclusions obtained in our study about the physicochemical properties of copper(ii) sulfate solutions in water and other solvents, with the reported literature, has also been performed for the supportive evidence. The trends of limiting apparent molar expansibilities (*E*^0^_ϕ_) and the isobaric thermal expansion coefficient (*α*_p_) concerning temperature are consistent with the conclusions obtained from the value of Helper's constant.

## Conflicts of interest

I hereby declare that there is no conflict of interest regarding this manuscript.

## Data Availability

The authors declare that the data related to the manuscript entitled “Ionic interactions of copper sulfate in water, aqueous maltose, and aqueous lactose at different temperatures: a volumetric and thermodynamic study” will be made available on request.
